# FBX8 promotes metastatic dormancy of colorectal cancer in liver

**DOI:** 10.1038/s41419-020-02870-7

**Published:** 2020-08-14

**Authors:** Xiaohui Zhu, Feifei Wang, Xuehui Wu, Zhou Li, Zhizhi Wang, Xiaoli Ren, Yangshu Zhou, Fuyao Song, Yunshi Liang, Zhicheng Zeng, Wangjun Liao, Yanqing Ding, Wenting Liao, Li Liang

**Affiliations:** 1 Department of Pathology, Nanfang Hospital and Basic Medical College, Southern Medical University, Guangzhou, 510515 Guangdong Province People’s Republic of China; 2 Guangdong Province Key Laboratory of Molecular Tumor Pathology, 510515 Guangzhou, Guangdong Province People’s Republic of China; 3 The First Clinical Medical Department, Southern Medical University, 510515 Guangzhou, Guangdong Province People’s Republic of China; 4 Department of Thoracic Surgery, Nanfang Hospital, Southern Medical University, 510515 Guangzhou, Guangdong Province People’s Republic of China; 5 Department of Oncology, Nanfang Hospital, Southern Medical University, 510515 Guangzhou, Guangdong Province People’s Republic of China

**Keywords:** Colorectal cancer, Cell growth

## Abstract

Patients with colorectal cancer (CRC) often develop malignant regrowth of metastatic dormant tumor cells in liver years after primary treatment. FBX8 is involved in suppressing tumor metastasis. Short-term chemotherapy experiments and liver metastasis mice model of orthotopic injection into the cecum were performed to construct the dormant models. GST-pull-down assay, Co-IP and immunofluorescence were used to confirm the bindings among FBX8 and its substrates. FBX8 upregulated the expression of epithelial and stemness markers, while downregulated the expression of mesenchymal and proliferative markers associated with tumor cell dormancy. FBX8 promoted the maintenance of metastatic dormancy of CRC cells. Mechanistically, FBX8 directly bound to HIF-1α, CDK4 and C-myc through its Sec7 domain and led to the ubiquitin degradation of these proteins, thereby inhibiting cell cycle progression, proliferation, angiogenesis, and metastasis. Clinically, FBX8 expression was negatively correlated with the HIF-1α, CDK4, and c-Myc in CRC tissues. Our study reveals a novel mechanism of FBX8 in regulating tumor metastatic dormancy in liver and provides new strategies for the treatment of CRC metastasis.

## Introduction

Colorectal cancer (CRC) is one of the most common malignant tumors. However, 30 to 40% of the patients will develop local regional recurrence or distant metastasis^1^. The liver is the most common site of metastatic recurrence in patients with CRC^2^.

In the process of distant metastasis of tumor cells, the primary tumor cells invade the blood vessels of surrounding tissues and then enter the blood as disseminated tumor cells (DTCs)^3^. DTCs penetrate blood vessels and reach target organs to form micro-metastases (1–2 mm^3^). Instead of proliferating quickly to form visible metastases, the metastatic tumor cells often choose to adapt to the new microenvironment in a non-amplification state for a long time and enter cellular dormancy. Subsequently, dormant tumor cells are activated and proliferate rapidly because of effects from target organ microenvironment, leading to clinically visible metastatic lesions^4^.

Tumor dormancy is a state in which tumor cells exist in the host body and go undetected for a long time. It is clinically common in patients after primary tumor resection, but will eventually produce overt local or metastatic recurrence years or decades after treatment^5^. Tumor dormancy has been observed in many types of solid tumors, including breast cancer, prostate cancer, and melanoma^6^. Currently, there are no specific markers for dormant tumor cells, but often with features of cell cycle arrest, slow proliferation or quiescent behaviors, stemness and with ability to escape frontline treatment and host immunity^7^. However, liver metastatic cell dormancy from CRC and its underlying molecular mechanism have not been reported. Therefore, we set to investigate this process by constructing multiple tumor dormancy models and explore the molecular mechanism of the dormancy of liver metastatic cells from CRC.

FBX8 is a new member of the F-box protein family with an F-box and Sec7 domain. Upregulation of FBX8 in breast cancer cells can inhibit the invasion of tumor cells mediated by ARF6^8^. FBX8 is found to be a new C-myc binding protein, which promotes tumor cell invasion by inhibiting the function of FBX8^9^. Low level of FBX8 expression is associated with glioma grading and poor prognosis^10^. In addition, our previous studies have established the relationship between FBX8 and tumor metastasis in liver cancer^11^ and gastric cancer^12^. Our study also found that FBX8 inhibits invasion and metastasis of CRC by degradation of m-TOR under the transcriptional regulation of miR-223^13^. In the current study, we set to investigate the role and mechanism of FBX8 in regulating the metastatic dormancy of CRC in liver.

## Materials and methods

### In vitro chemotherapy dormancy model of CRC cells

HT29 cells were cultured in a 96-well plate. Oxaliplatin and 5-FU were used to treat HT29 cells at a concentration of 1, 5, 10, and 20 μmol/L 48 h. After drug treatment, cell proliferation was performed by CCK8 and EDU assays. The drug concentration with effective reduced cell proliferation and good cell viability was selected as the appropriate concentration of chemotherapy.

Following drug treatment, HT29 cells were transduced with lentivirus repressing FBX8 and vector overexpressing FBX8. The control group of over-expression FBX8 was treated with MG132 and the proliferation of cells was detected by CCK8 and EDU. The expression of FBX8, HIF-1α, C-Myc, and CDK4 were analyzed by western blot.

### Construction of liver metastasis dormancy model of CRC in nude mice

In all, 2 × 10^6^ CRC cell line HT29 were mixed with Matrigel Matrix (1:2) before being injected into the cecum of nude mice. Mice were sacrificed daily for the growth observation of liver metastatic tumors. The investigator was blinded to the group allocation when measuring tumor volue. The growth curves of liver metastases in mice were drawn according to the size of the tumor under microscope.

### Construction of a tracer model for liver metastasis dormancy of CRC in C57 mice

In all, 1 × 10^7^ CMT93 cells transfected with luciferase were injected into the cecum of C57 mice. Caliper IVIS Lumina II was used to observe the tumor formation in the cecum and liver. Before the tumor formed visible liver metastases (7 days after injection), the primary tumor in the cecum was surgically removed, and then one group of mice were injected intra-peritoneally with MG132 for 7 consecutive days (2 mg/kg) on day 14. After intraperitoneal injection of luciferase substrates obout every 15 days, Caliper IVIS Lumina II was used to observe the liver metastasis, and the fluorescence value of liver metastasis was recorded. The growth curve of liver metastases in mice was drawn according to the fluorescence value of metastatic tumors.

### Immunofluorescence

Cells cultured in culture dishes were fixed with 4% paraformaldehyde (PFA) for 5 min at room temperature (RT), followed by washing with PBS. Cells were then permeabilized with 0.25% Triton X-100 in PBS for 5 min, washed again with PBS before being blocked with Goat Serum for 30 min. Finally, cells were incubated with primary antibodies diluted in blocking buffer for 1 h at RT in a dark humid chamber. After washing with PBS, Cells were incubated with diluted secondary antibody for 1 h at RT before being counterstained with DAPI. Each experiment was repeated three times.

### Co-immunoprecipitation (Co-IP)

SW620, SW480, and HT29 cell extracts were incubated 2 h at 4 °C with IgG and protein A + G-Agarose to get rid of non-specific binding. Primary antibodies against FBX8, HIF-1α, CDK4, and C-Myc was then added to separate cell extract tubes for incubation at 4 °C overnight. The protein A/G-agarose was collected by centrifugation. Immunoprecipitated proteins were analyzed by sodium dodecyl sulfate polyacrylamide gel electrophoresis ((SDS-PAGE) 10%, Minigel) at 100 V for 1.5 h. Membranes were blocked first before FBX8 and mTOR antibodies were added for incubation at 4 °C overnight. The secondary antibodies were then incubated for 1 h at room temperature before final detection with enhanced chemiluminescence (PerkinElmer Life Sciences).

### Glutathione S-transferase (GST) pull-down assay

The interaction of truncated FBX8 with HIF-1α, CDK4, and C-Myc was examined in HT29 cells by GST-mediated pull-down assays (Thermo Scientific, Rockford, IL). Recombinant FBX8 protein was expressed and purified. Purified GST-FBX8, GST-FBX-Sec7 fragments were bound to glutathione resin as a GST-fusion protein and incubated with GSTP1 at 4 °C for 2 h. After extensive washing with assay buffer, the complex was eluted with 5 mM reduced glutathione and the bound protein complexes were disrupted. Then, the proteins were separated on SDS-PAGE for western blotting analysis.

### Immunoprecipitation

In brief, the vector containing cytomegalovirus (CMV) promoter driven pReceiver-M06-HA-ubiquitin was generated and then transfected into SW620/NC, SW620/shFBX8, SW480/FBX8, and SW480/MOCK cells. After 48 h, the extracts were prepared in lysis buffer. Lysates were precleared with protein A/G-sepharose beads (GE Healthcare) and then incubated with anti-HA overnight at 4 °C. After that, the beads were washed three times with lysis buffer and separated out by centrifugation at 4 °C, 2500 r.p.m. The precipitates were subjected to immunoblotting.

### Tube formation assay

Matrigel matrix (Corning) was plated in 50 μ-Slide Angiogenesis ibiTreat plate (ibidi), incubated at 37 °C for 30 min to allow the matrigel to polymerize. The HUVECs were seeded on the matrigel-coated well, SW620/NC, SW620/shFBX8, SW480/MOCK, SW480/FBX8, SW480/FBX8/HIF-1α, HT29/MOCK, HT29/FBX8 and HT29/FBX8/HIF-1α conditioned medium were added, then the plate was incubated at 37 °C in 5% CO_2_ humidified atmosphere. Tube formation was observed at 6 h with microscope. The tube formation ability was determined by measuring the number of tubes. Each experiment was repeated three times.

## Results

### FBX8 is correlated with tumor dormancy in CRC

**Fig. 1:**
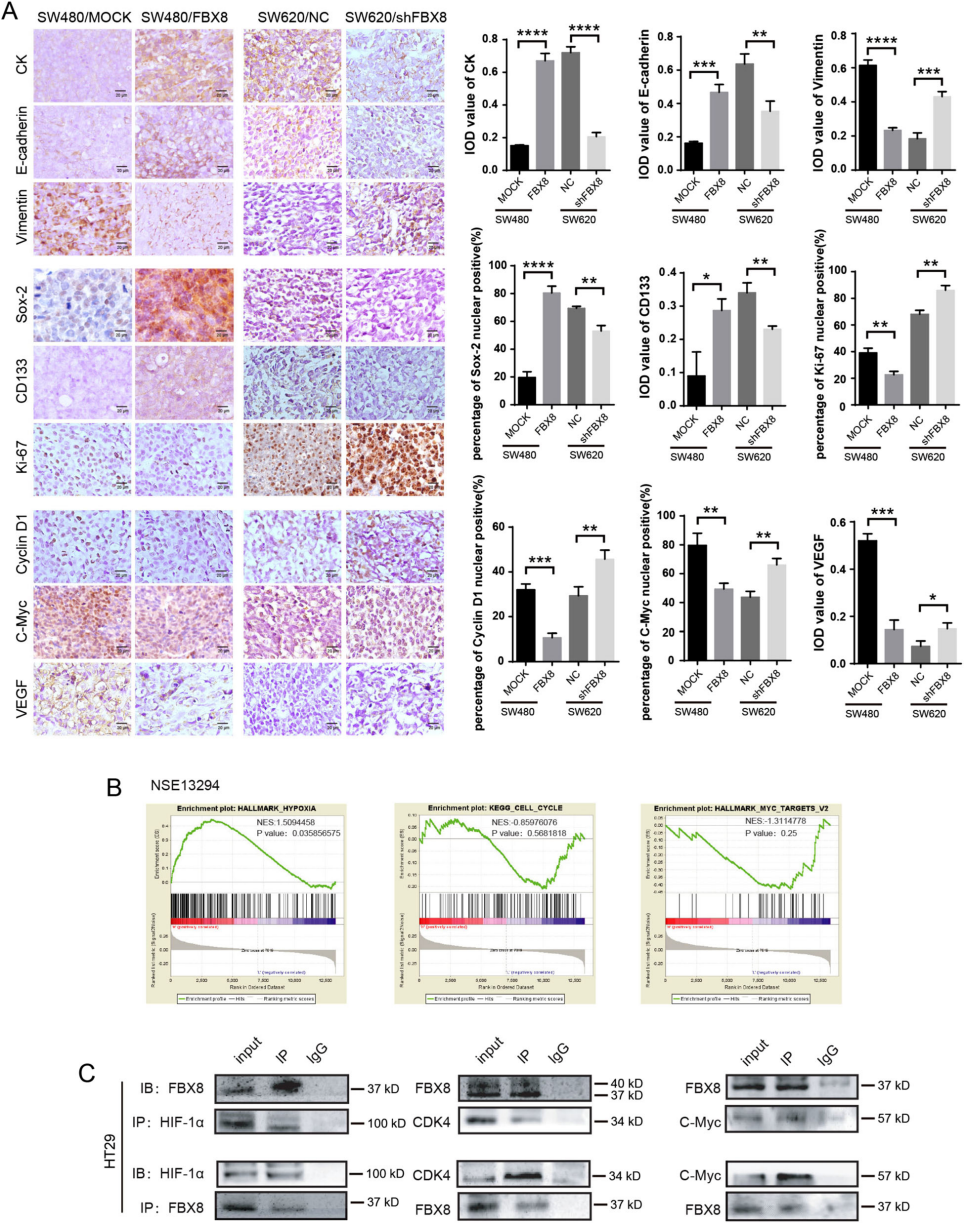
FBX8 regulates dormancy-related genes in CRC cells. **a** IHC staining of CK, E-cadherin, Vimentin, Sox-2, CD133, Ki-67, cyclin-D1, C-Myc, and VEGF in subcutaneous tumors of SW480/MOCK, SW480/FBX8, SW620/NC, and SW620/shFBX8 cells. Data represent mean ± SD. Scale bars represent 20 μm. **b** The Gene enrichment analysis of FBX8 in CRC by GSEA(NSE13294). **c** The screening of FBX8-interacting proteins by Co-IP assay in HT29 cells. *P < 0.05, **P < 0.01, ***P < 0.001, ****P < 0.0001.

As there are no specific markers for dormant tumor cells, we selected genes that were related to cell cycle arrest, proliferation, stemness, epithelial–mesenchymal transition (EMT), and hypoxia regulations as indicators of tumor dormancy in the current study. To test whether aberrant regulation of FBX8 was involved in tumor dormancy, we examined the expression of cell dormancy-related markers (including CK, E-cadherin, Sox-2, and CD133) and cell dormancy activation related markers (including Vimentin, cyclin-D1, Ki-67, C-myc, and VEGF) in the subcutaneous tumors formed by previously established SW480/MOCK, SW480/FBX8, SW620/NC, and SW620/shFBX8 stable cell lines^13^ in nude mice. As shown in (*P* < 0.05, *P* < 0.01, *P* < 0.001, *P* < 0.0001, Fig. 1a), the expression of cell dormancy-related markers, including CK, E-cadherin, Sox-2, and CD133 was significantly upregulated in the FBX8-overexpressed tumors as compared with control tumors. In contrast, the expression of cell dormancy activation related markers, including Vimentin, cyclinD1, Ki-67, C-myc, as well as VEGF was downregulated in the tumors with FBX8-knockdown. These results indicate that FBX8 is associated with tumor cell dormancy. Gene enrichment analysis (GSEA) was performed using human CRC datasets to evaluate whether FBX8 was associated with tumor dormancy in human CRC tumors. The results showed that the expression of FBX8 was significantly correlated with dormancy-related hypoxia, cell cycle, and Myc pathways (Fig. 1b). Co-IP revealed that FBX8 could directly interact with HIF-1α, CDK4 and C-Myc (Fig. 1c and Supplementary Fig. S1). These data suggest that FBX8 is involved in the modulation of tumor dormancy by regulating HIF-1α, CDK4, and c-Myc proteins.

### FBX8 promotes tumor dormancy in vitro

**Fig. 2:**
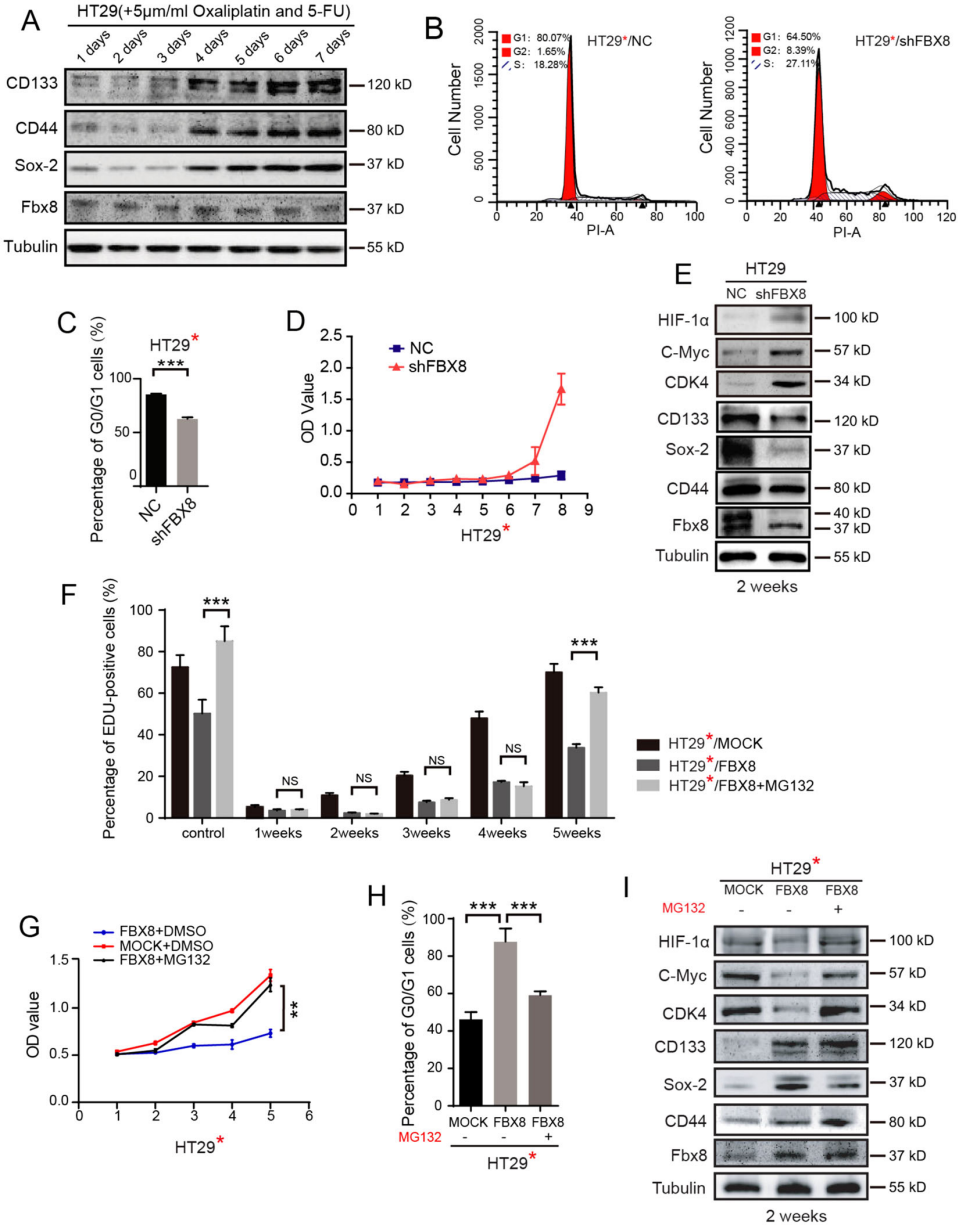
FBX8 promotes domancy of CRC in vitro. **a** Western blot analysis was performed for CD44, SOX-2, CD133, and FBX8 in HT29 cells with 5 μmol/L concentrations of oxaliplatin and 5-FU combined chemotherapy, normalized to tubulin. **b**, **c** Effects of FBX8 on HT29 cells proliferation at 5 weeks by cell cycle assay. Data represent mean ± SD. **d** Effects of FBX8 on cell proliferation by CCK8 assay. Data represent mean ± SD. **e** Western blot analysis was performed for HIF-1α, C-Myc, CDK4, CD44, SOX-2, CD133, and FBX8 in HT29 cells with 5 μmol/L concentrations of oxaliplatin and 5-FU combined chemotherapy, normalized to tubulin. **f** Edu analysis was performed for the proliferation of HT29 cells with 5 μmol/L concentrations of oxaliplatin and 5-FU combined chemotherapy in vitro. Data represent mean ± SD. **g** Effects of FBX8 on HT29 cells proliferation at 5 weeks by CCK8 assay. Data represent mean ± SD. **h** Effects of FBX8 on HT29 cells proliferation at 5 weeks by cell cycle assay. Data represent mean ± SD. **i** Western blot analysis was performed for HIF-1α, C-Myc, CDK4, CD44, SOX-2, CD133, and FBX8 in HT29 cells with 5 μmol/L concentrations of oxaliplatin and 5-FU combined chemotherapy, normalized to tubulin. *P < 0.05, **P < 0.01, ***P < 0.001.

To test whether FBX8 can regulate tumor dormancy in CRC, we sort to find an appropriate cell lines to establish an in vitro cell culture model of tumor dormancy among 12 CRC cells lines by examining the expression of stem cell markers CD44, CD133, SOX-2, SOX-9, and FBX8-interacting proteins HIF-1α, C-Myc, and CDK4^14,15^. Among these cell lines, HT29 cell line was highly expressed with FBX8, SOX-2 and SOX-2, and CD44, while quite weakly expressed with CDK4, HIF-1α, and c-Myc (Supplementary Fig. S2A), indicating that HT29 cell line was an appropriate model to study tumor dormancy. Both Oxaliplatin and 5-FU have been used to induce tumor cells to enter cellular quiescence, which is a hallmark of clinical tumor dormancy^16^. To establish an in vitro tumor dormancy model, we performed short-term acute chemotherapy on HT29 cells to induce dormancy^17^. HT29 cells were treated with Oxaliplatin and 5-FU with four different concentrations at 1, 5, 10, and 20 μmol/L. All drug concentrations except 1 μmol/L successfully induced dramatically reduced cell proliferation at day 7 as compared to control cells (*P* < 0.01, *P* < 0.001, Supplementary Fig. S2B, C). In addition, Oxaliplatin and 5-FU treatment at 5, 10, and 20 μmol/L led to significant increased cell numbers that enter G0/G1 phase (Supplementary Fig. S2D; *P* < 0.001, Supplementary Fig. S2E). Based on these results, we therefore chose 5 μmol/L as an ideal concentration to induce tumor dormancy in HT29 cells. Upon Oxaliplatin/5-FU treatment, the expression of SOX-2, CD44, and CD133 began to rise on day 4 and maintained at high-expression levels at day 5, 6, and 7 (Fig. 2a). These results suggest that HT29 cells enter dormancy on day 4 after treatment with Oxaliplatin/5-FU.

To study the role of FBX8 in tumor dormancy, the FBX8 was stably knockdown in HT29 cells followed by Oxaliplatin/5-FU treatment. Knockdown of FBX8 significantly reduced the cell numbers that enter G0/G1 phase (Fig. 2b; *P* < 0.001, Fig. 2c) and increased the cell proliferation rate (*P* < 0.01, Fig. 2d). In addition, the dormancy activation related markers (HIF-1α, CDK4, and C-Myc) were upregulated, while the dormancy-related cell stem markers (SOX-2, CD44, and CD133) were downregulated in the HT29/shFBX8 cells at 2 weeks after Oxaliplatin/5-FU treatment (Fig. 2e). These results indicate that FBX8 is necessary for the maintenance of tumor dormancy in HT29 cells.

To further confirm the role of FBX8 in tumor dormancy, HT29 cells were stably overexpressed with FBX8 and followed by Oxaliplatin/5-FU treatment. Proteasome inhibitor MG132 (5 µM/L) was applied to suppress the degradation of ubiquitination substrate of FBX8. Overexpression of FBX8 significantly suppressed the proliferation of HT29 cells, while this effect could be rescued by MG132 treatment (*P* < 0.01, *P* < 0.001, Fig. 2f, g and Supplementary Fig. S3A). Meanwhile, overexpression of FBX8 dramatically increased the proportion of G0/G1 phase cells, which could also be abolished by MG132 treatment (*P* < 0.001, Fig. 2h and Supplementary Fig. S3B). In addition, overexpression of FBX8 significantly decreased the expression of dormancy activation related markers (HIF-1α, CDK4, and C-Myc), while increased the expression of dormancy-related cell stem markers (including SOX-2, CD44, and CD133) (Fig. 2i). Moreover, the downregulation of dormancy activation markers and upregulation of dormancy-related cell stem markers were diminished by MG132 treatment (Fig. 2i). Taken together, these above results indicate that FBX8 promotes dormancy of CRC cells in vitro, and this process can be inhibited by MG132.

### FBX8 promotes metastatic dormancy of CRC in vivo

**Fig. 3:**
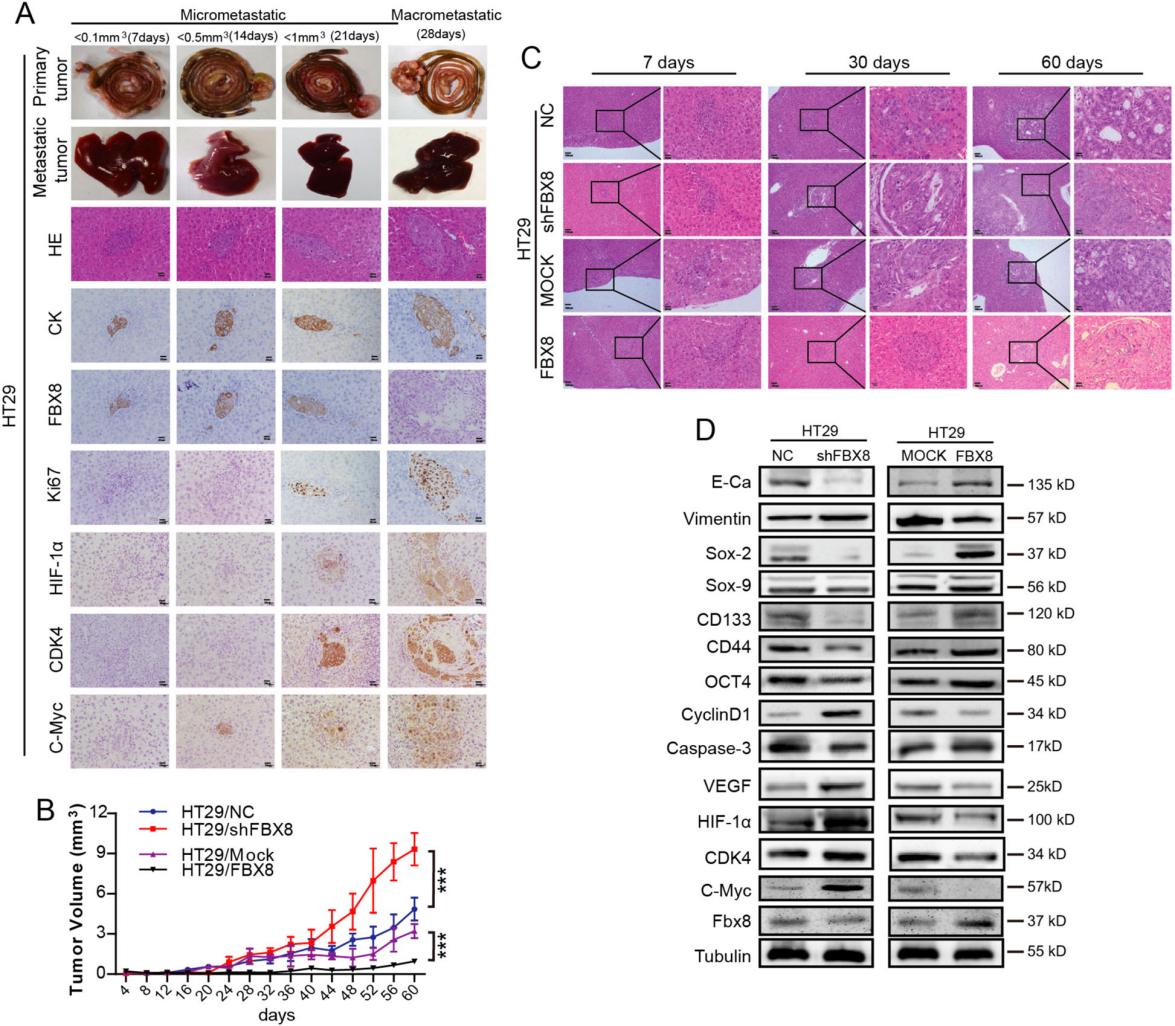
FBX8 promotes domancy of CRC in nude mice. **a** The expressions of CK, FBX8, Ki-67, HIF-1α, CDK4, and C-Myc in liver metastatic tumors detected by IHC. Scale bars represent 20 μm. **b** The growth curve of Liver metastases in nude mice. Data represent mean ± SD. **c** The metastatic tumors from HT29 cell lines in liver at different points in time. Scale bars represent 100 μm or 20 μm. **d** Western blot analysis was performed for E-cadherin, Vimentin, SOX-2, SOX-9, CD133, CD44, OCT-4, Cyclin-D1, Caspase-3, VEGF, HIF-1α, CDK4, C-Myc, and FBX8 in HT29/NC, HT29/shFBX8, HT29/MOCK, and HT29/FBX8 tumor in situ of cecum normalized to tubulin. *P < 0.05, **P < 0.01, ***P < 0.001.

Currently, there are few animal models established to be able to faithfully recapitulate metastatic dormancy. In order to investigate tumor dormancy in liver metastasis of CRC, we established an in vivo dormancy model using orthotopic injection into the cecum of nude mice using HT29 cells. Microscopic metastases (<1 mm^3^) were observed from day 7 after injection by collecting tumors and observing the H&E sections daily. The metastases grew slowly in the liver before day 28 (Fig. 3a and Supplementary Fig. S4A). Surprisingly, the metastatic tumor cells began to grow rapidly and formed macroscopic metastases (>1 mm^3^) at liver surface on day 28. Therefore, we defined liver metastasis of CRC in mice with a volume less than 1 mm^3^ as micrometastasis, and greater than or equal to 1 mm^3^ as macrometastasis. The interval between the microscopic appearance of microscopic metastases on day 7 and the rapid growth of metastases on day 28 was the dormant phase of metastatic tumors. (Fig. 3a). As expected, the expression of FBX8 and CK were dramatically downregulated, while the Ki-67, HIF-1α, CDK4, and C-Myc were significantly upregulated in the metastatic tumor cells collected at day 28 as compared to those collected at days 7, 14, and 21 (Fig. 3a). This data indicates that metastatic tumor cells in the liver stay in a dormant status with high level of FBX8 expression before day 28.

Further study showed that overexpression of FBX8 significantly suppressed (*P* < 0.001, Supplementary Fig. S4B, C), while knock down of FBX8 greatly increased the tumor growth in situ (*P* < 0.001, Fig. 3b) and metastasis in the liver (Fig. 3c). In addition, over-expression of FBX8 significantly prolonged, while knockdown of FBX8 shortened the dormant phase of CRC tumors (Fig. 3c). Moreover, overexpression of FBX8 increased, while knockdown of FBX8 decreased the expression of E-cadherin, SOX-2, SOX-9, CD133, CD44, OCT-4, and Caspase-3 expression in tumors. On the contrary, overexpression of FBX8 decreased, while knockdown of FBX8 increased the expression of Vimentin, Cyclin-D1, VEGF, HIF-1α, CDK4, and C-Myc (Fig. 3d). These results indicate that FBX8 significantly promotes tumor dormancy of metastatic CRC cells in the liver.

### FBX8 inhibits tumor relapse after surgery

**Fig. 4:**
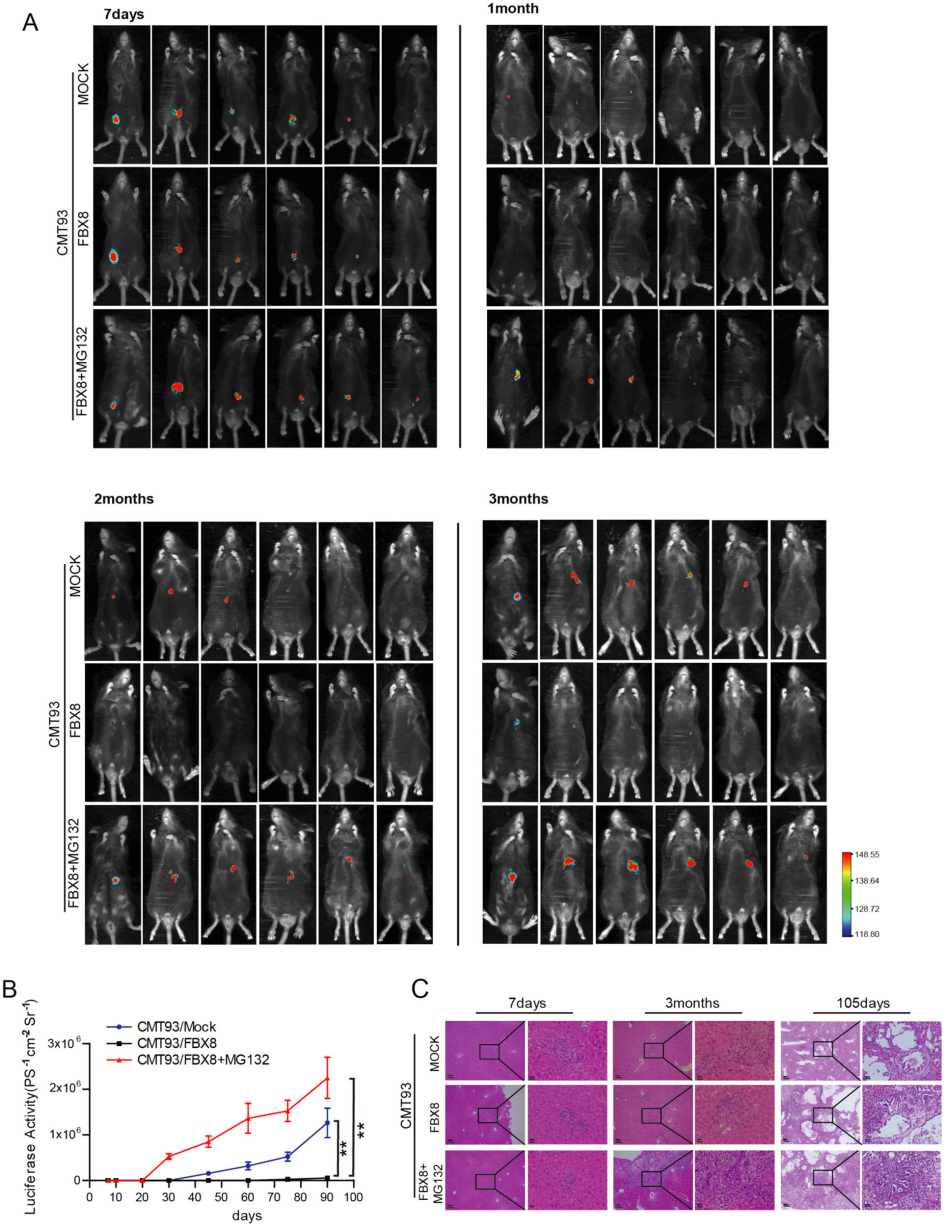
FBX8 inhibits tumor relapse after surgery. **a** Luciferase detection for tumor growth in C57 mice at 7 day, 1 month, 2 months, and 3 months. **b** The growth curve of Liver metastases in C57 mice. Data represent mean ± SD. **c** The HE stains of liver metastatic tumor in C57 mice. Scale bars represent 100 μm or 20 μm. *P < 0.05, **P < 0.01.

Tumor dormancy may contribute to tumor relapse after surgery. To mimic the relapse of human CRC after surgery, we generated a stable CMT93 cell line transduced with both a luciferase and FBX8 mini-genes (CMT93/luc/FBX8) (*P* < 0.001, Supplementary Fig. S5A, B). CMT93/luc/FBX8 cells were orthotopically injected into the cecum of C57 mouse and primary tumor formation was observed at day 7 after injection (Supplementary Fig. S5C). On day 14 after injection, mice with CMT93/luc/FBX8 tumors were treated with MG132 intra-peritoneally to monitor tumor growth and metastasis under an animal live imaging system. The results showed that FBX8 over-expression drastically suppressed the formation of metastasis in the liver (Fig. 4a; *P* < 0.01, Fig. 4b, c), and no visible metastasis occurred until the 105th day. Conversely, MG132 treatment significantly increases the formation of liver metastasis from CMT93/luc/FBX8 tumors in mice (Fig. 4a–c), the macrometastasis occurred on day 30. This result further supported the notion that over-expression of FBX8 promotes the dormancy of liver metastasis cells. Treatment of ubiquitin-proteasome inhibitor MG132 can effectively abolish of the dormancy and re-activated the dormant cell growth in the liver.

### FBX8 promotes the degradation of HIF-1α, CDK4, and C-Myc proteins

**Fig. 5:**
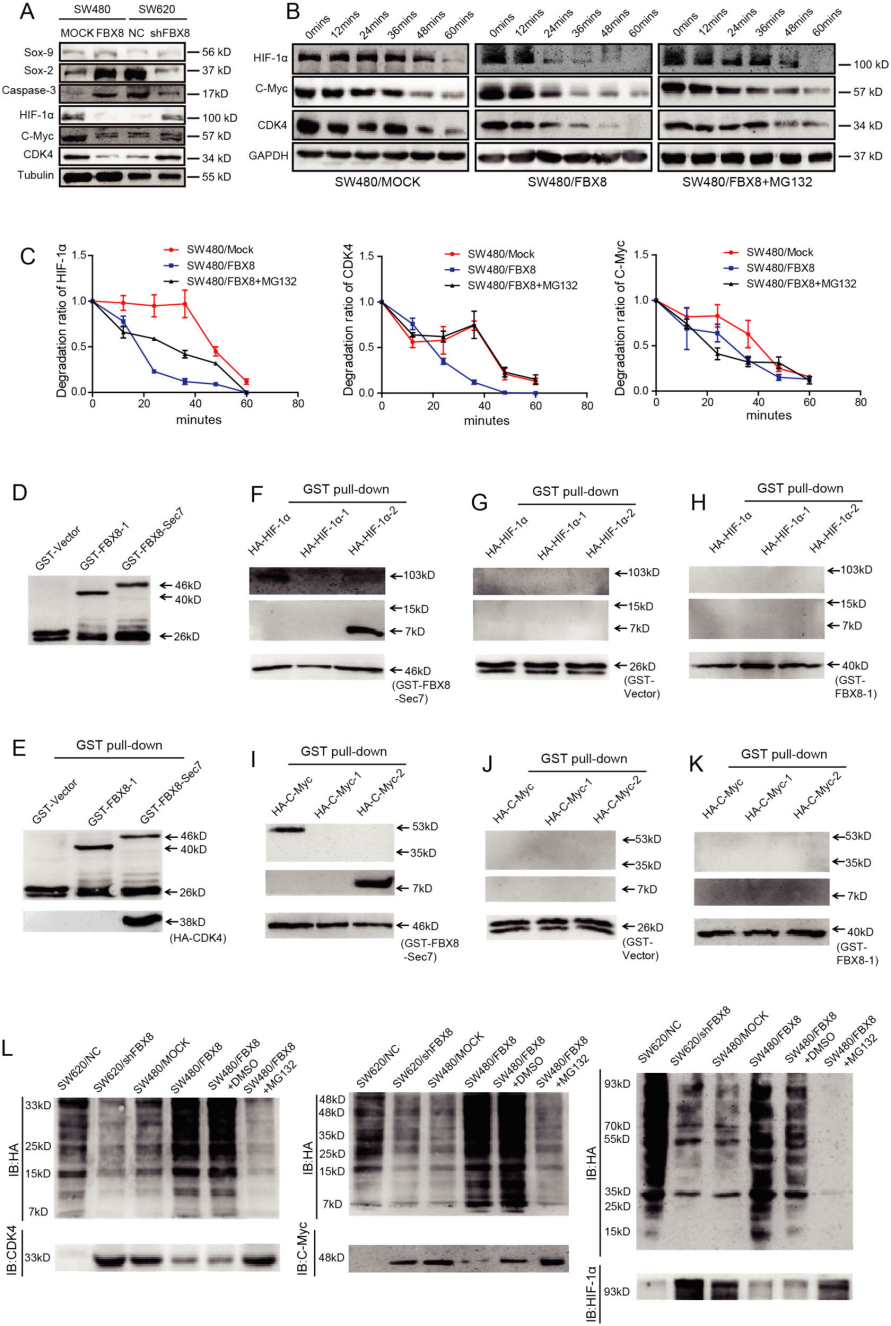
FBX8 targets HIF-1α, CDK4, and C-Myc for ubiquitin-mediated degradation. **a** Western blot analysis was performed for SOX-9, SOX-2, Caspase-3, HIF-1α, C-myc, and CDK4 in subcutaneous tumors of SW480/MOCK, SW480/FBX8, SW620/NC, and SW620/shFBX8 cells, normalized to tubulin. **b**, **c** The half-life of HIF-1α, CDK4, and C-Myc were detected by a cycloheximide chase assay. **d** The expression of GST in *E coli*. detected by western blot. **e**–**k** *E coli*. transfected with an FBX8-expressing construct and GST-tagged IP of FBX8-Sec7 and FBX8-1 identified HA-HIF-1α-2, CDK4, and HA-C-Myc-2 as a binding domain. **l** SW620/NC, SW620/shFBX8, SW480/MOCK, SW480/FBX8, and SW480/FBX8 cells with MG132 treatment were co-transfected with plasmids expressing HA-Ubiquitin, and IP of HIF-1α, CDK4, and C-Myc followed by immunoblot analysis of HA-ubiquitin.

Since FBX8 could interact with HIF-1α, CDK4, and C-Myc proteins directly and FBX8 ubiquitinates and degradates target proteins, it is possible that FBX8 may promote the degradation of these proteins. Immunofluorescence staining showed that FBX8 co-localized with HIF-1α, CDK4, and C-Myc in CRC cells. In addition, MG132 treatment increased this kind of co-localization (Supplementary Fig. S6A–C). Western Blot showed that overexpression of FBX8 downregulated, while knockdown of FBX8 upregulated the expression of HIF-1α, CDK4, and C-Myc (Fig. 5a). We next examined effects of FBX8 on the half-life of HIF-1α, CDK4, and C-Myc proteins. Over-expression of FBX8 significantly decreased the half-life of HIF-1α, CDK4, or C-Myc protein, while this effect can be rescued by MG132 treatment (Fig. 5b, c). Mutant constructs were generated and GST-pull-down assay were performed to confirm the binding between FBX8 and HIF-1α, C-Myc, and CDK4 (Fig. 5d), showing that FBX-Sec7 mutant (11 FBX8 32-1700a) could directly interact with HA-HIF-1α, HA-HIF-1α-2, HA-CDK4, HA-C-myc, or HA-C-myc-2 (Fig. 5e–k). Ubiquitination degradation assay showed that knockdown of FBX8 significantly reduced, whereas overexpression of FBX8 dramatically increased the expression of ubiquitin small molecules (Fig. 5l). Importantly, MG132 treatment significantly inhibited the expression of ubiquitin small molecules induced by FBX8 overexpression (Fig. 5l). These results suggest that FBX8 promotes HIF-1α, CDK4, and C-Myc degradation through ubiquitination. Taken together, the data indicate that FBX8 promotes the degradation of HIF-1α, CDK4, and C-Myc through the ubiquitin-proteasome pathway.

### FBX8 induces stemness, and suppresses proliferation, cell cycle progress, and angiogenesis through targeting C-Myc, CDK4, and HIF-1α, respectively, in CRC cells

**Fig. 6:**
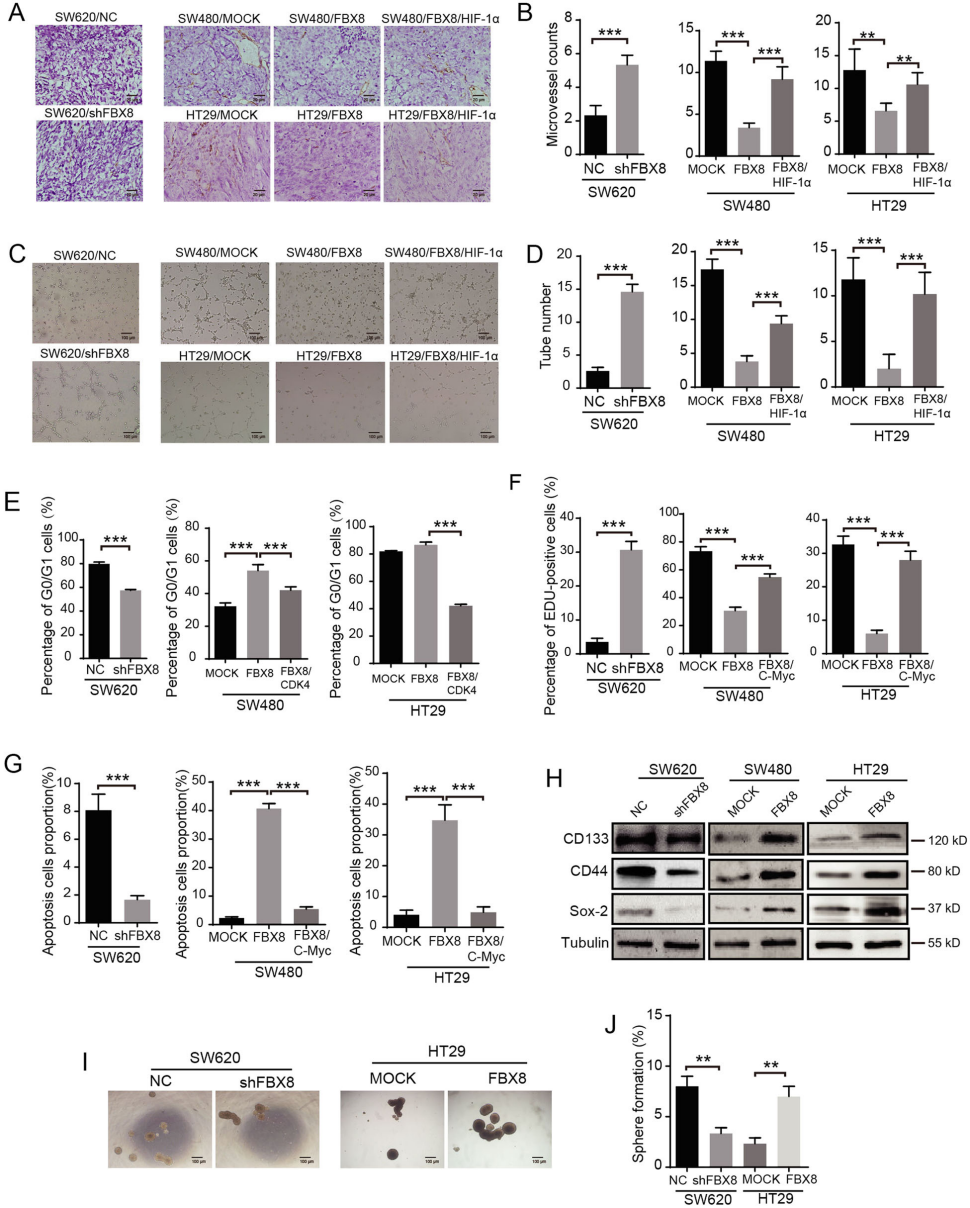
FBX8 targets HIF-1α, CDK4, and C-Myc to regulates angiogenesis, cell cycle, and proliferation of CRC cells, respectively. **a**, **b** Effects of FBX8 on microvascular formation ability in SW620, SW480, and HT29 cells subcutaneous tumor formation in nude mice detected by IHC. Scale bars represent 20 μm. Data represent mean ± SD. **c**, **d** Effects of FBX8 on tube formation in SW620, SW480, and HT29 cells. Scale bars represent 100 μm. Data represent mean ± SD. **e** The cell cycle of SW620, SW480, and HT29 cell lines. Data represent mean ± SD. **f** The proliferation of SW620, SW480, and HT29 in vitro detected by Edu. **g** Effects of FBX8 on apoptosis in SW620, SW480, and HT29 cell lines. Data represent mean ± SD. **h** Western blot analyses were performed for CD44, SOX-2, and CD133 in SW620, SW480, and HT29 cell lines. **i**, **j** Effects of FBX8 on sphere formation rate in SW620 and HT29 cells. Scale bars represent 100 μm. Data represent mean ± SD. *P < 0.05, **P < 0.01, ***P < 0.001.

It has been reported that HIF-1α, as nuclear transcription factor regulating tumor cells to adapt to anoxia, plays an important role in the regulation of tumor cell neo-vascularization^18^. CDK4 can regulate cell cycle^19^. C-Myc regulates transcription factors and feature in cell proliferation, transformation, and apoptosis. Therefore, we examined the effects of FBX8/HIF-1α, FBX8/CDK4, and FBX8/C-Myc, on tumor angiogenesis, cell cycle progression, cell proliferation, and stem cell potential, respectively (Supplementary Fig. S7A). We performed microvessel density formation assay and counted number of CD34-positive blood vessels on subcutaneous tumor tissue of nude mice, showing that knockdown of FBX8 increased the number of micro-angiogenesis in subcutaneous tumors and HUVEC cells lumen formation in vitro (Fig. 6a; *P* < 0.01, *P* < 0.001, Fig. 6b, c; *P* < 0.001, Fig. 6d). However, overexpression of FBX8 significantly reduced, while reintroduction of HIF-1α dramatically promoted angiogenesis both in vitro and in vivo (Fig. 6a–d). In addition, knockdown of FBX8 significantly reduced the proportion of G0/G1 phase cells. While the ratio of G0/G1 phase cells in the FBX8 overexpressing group was higher than that in the control group in SW480 and HT29 cells. However, further overexpression of CDK4 obviously decreased the proportion of G0/G1 phase cells in FBX8 overexpressing cells (*P* < 0.001, Fig. 6e and Supplementary Fig. S7B). The above results indicate that FBX8 induces cycle arrest of CRC cells by suppressing CDK4. Moreover, EDU assays revealed that knockdown of FBX8 significantly increased the cell proliferation. However, overexpression of FBX8 significantly decreased, while transfection of c-Myc dramatically promoted proliferation (*P* < 0.001, Fig. 6f and Supplementary Fig. S7C). Apoptosis assay showed that knockdown of FBX8 significantly inhibited apoptosis. However, overexpression of FBX8 significantly induced, while further overexpression of c-Myc dramatically inhibited proliferation (*P* < 0.001, Fig. 6g and Supplementary Fig. S7D). These results indicate that FBX8 can inhibit proliferation, while promote apoptosis of tumor cells by suppressing C-Myc. In addition, overexpression of FBX8 can upregulate the expression of stemness markers (Fig. 6h) and promote spherulation (Fig. 6i; *P* < 0.01, Fig. 6j) in CRC cells. Taken together, FBX8 regulates the biological function dormant tumor cells by maintainning tumor cell stemness and targeting C-Myc, CDK4, and HIF-1α.

### FBX8 expression is negatively correlated with HIF-1α, CDK4, and c-Myc in human CRC samples

**Fig. 7:**
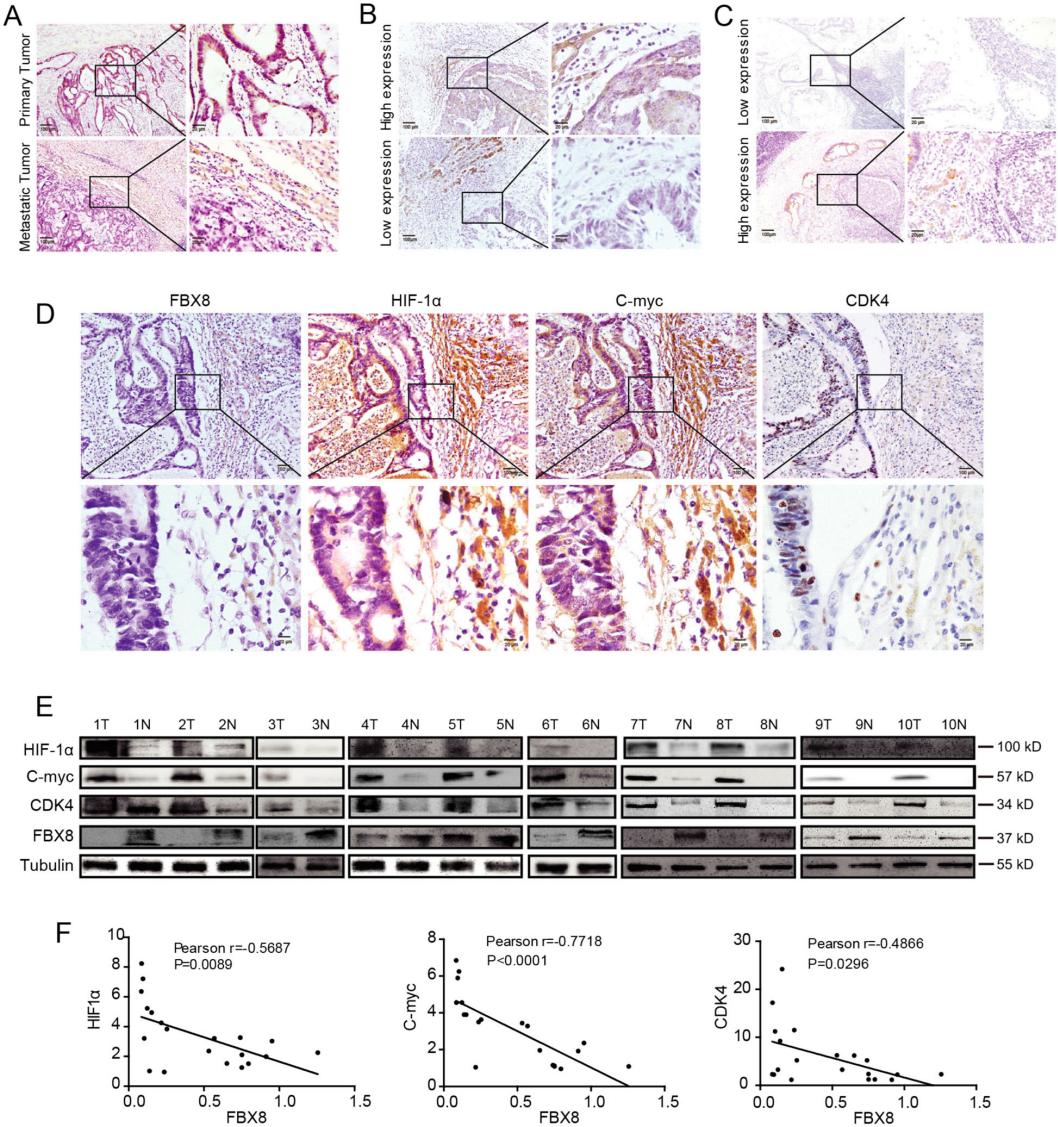
FBX8 expression is negatively correlated with HIF-1α, CDK4, and C-Myc in CRC. **a** IHC staining of FBX8 protein in primary tumors and liver metastatic tumors tissues. (*n* = 91). **b** IHC staining of FBX8 in lymph node metastasis of CRC tissues (*n* = 77). **c** IHC staining of FBX8 protein in liver metastatic tumors tissues. **d** IHC staining of FBX8, HIF-1α, CDK4, and C-Myc protein in liver metastasis tissues. Scale bars represent 100 μm or 20 μm. **b** Western blot analyses of FBX8, HIF-1α, CDK4, and C-Myc in ten paired CRC tissues and corresponding normal tissues. **c** The correlation analysis of FBX8 expression with HIF-1α, CDK4 and c-myc expression in fresh CRC paired tissues.

To determine the clinical relevance of FBX8 in CRC, IHC was performed using antibody against FBX8 in primary (91 cases) and liver metastases (77 cases) of CRC. The results showed that the expression of FBX8 in liver metastases was lower than that in primary cancer (Fig. 7a and Supplementary Fig. S8A). Kaplan–Meier survival analysis showed that prognosis of the group with high-FBX8 expression was better than that with low-FBX8 expression in patients with hepatic metastasis (Fig. 7b and Supplementary Fig. S8B). Meanwhile, FBX8 expression was closely associated with lymph node metastasis in CRC patients^13^. The high-expression group had a better prognosis than the FBX8 low-expression group (Fig. 7 and Supplementary Fig. S8C). Next, we used PROGgeneV2 (a tool that can be used to study prognostic implications of genes in various cancers) to analyze the recurrence-free survival rate of FBX8 in CRC and other tumors. The results showed that compared to low-FBX8 expression group, high-expression group had a better recurrence-free survival rate among CRC, breast cancer, and renal clear cell carcinoma (*P* < 0.05, *P* < 0.01, *P* < 0.05, Supplementary Fig. S8D–F). Combined with previous IHC results, FBX8 may be involved in the regulation of dormancy in CRC liver metastasis cells.

Next, we analyzed the correlation between FBX8 and HIF-1α, CDK4, and C-Myc in 91 cases of liver metastases from patients with CRC. The results showed that FBX8 expression was negatively correlated with HIF-1α, CDK4, and C-Myc in liver metastases (Fig. 7d and Table 1). In addition, the expression of FBX8, HIF-1α, CDK4, and C-Myc in 20 pairs of fresh CRC paired tissues was detected by western blot. The results showed that the expression of FBX8 in adjacent normal tissues was significantly higher than that in cancer tissues, while HIF-1α, CDK4, and C-Myc showed high expression in cancer tissues. The same results were obtained by reverse transcription PCR. Pearson correlation analysis showed that FBX8 was negatively correlated with HIF-1α, CDK4, and C-Myc mRNAs in fresh paired CRC tissues (Fig. 7e, f and Supplementary Fig. S8G, H). The above results indicate that FBX8 expression is negatively correlated with HIF-1α, CDK4, and C-Myc in human CRC liver metastasis and fresh paired CRC tissues.

**Table 1:** The correlation analysis of FBX8 expression with HIF-1α, CDK4, and C-myc in liver colorectal metastases.

Gene group	FBX8/HIF-1α	FBX8/CDK4	FBX8/C-myc
Spearman correlation	−0.711	−0.735	−0.631
*P*-value	<0.0001	<0.0001	<0.0001

## Discussion

In recent years, the incidence and mortality of CRC have shown a clear upward trend. Thanks to the advancement of novel diagnostic and therapeutic technologies, the overall survival rate has been greatly improved if relatively earlier diagnosis can be made. However, many patients have unfortunately developed to the metastasis stage at the time of diagnosis, especially liver metastasis, those patients miss the best treatment opportunity. After CRC cells metastasize to liver, they do not form macroscopic visible lesion right away. Instead, these cells enter a dormant period in the liver and become resistant to therapeutic interferences. It has been reported that a variety of mutations related to dormancy-related genes can affect the prognosis of CRC, while recurrence-free survival and overall survival rate are important indicators of evaluation^20^. The FBX8 gene is a member of the ubiquitin protease family and our previous studies have shown that low levels of FBX8 expression was associated with lower overall survival in patients with CRC^13^. Moreover, higher level of FBX8 expression in metastatic lymph node was associated with a good prognosis. We also performed survival analysis of FBX8 expression in liver metastatic sites from patients with CRC and similar results were obtained. At the same time, bioinformatics analysis showed that higher FBX8 expression was associated with high-recurrence-free survival of CRC, breast cancer, and renal clear cell carcinoma. There are many signaling pathways and genes involved in tumor dormancy. By reviewing previous literature, we found that tumor cell EMT^21^, tumor cell stem potential^22^, cell cycle^23^ and apoptosis^24^, proliferation and differentiation^25^, and tumor angiogenesis^26^ are all associated with tumor cell dormancy. Hence, we first used immunohistochemistry staining to evaluate tumor dormancy-related genetic markers in subcutaneous tumor tissues of nude mouse from previous animal assay in order to find downstream genes regulated by FBX8. The results showed that FBX8 could upregulate CK, E-cadherin, Sox-2, Caspase-3, and some other markers related to tumor cell dormancy. It also downregulated Vimentin, C-Myc, CDK4, Cyclin-D1, HIF-1α, and VEGF, which are known genetic markers related to dormancy activation of tumor cells. This suggests that FBX8 is involved in the regulation of a wide range of genes involved in tumor cell dormancy. Based on the above findings, we hypothesized that FBX8 may be involved in the dormancy of CRC at liver metastatic sites.

To further explore the interaction between FBX8 and these down-stream genes, we used gene enrichment analysis to enrich the FBX8 dormant-associated genes in CRC and found that FBX8 enriched hypoxia, cell cycle, and Myc-related genes. In addition to EMT, FBX8 interacted with genes involved in stem potential, cycle, apoptosis, proliferation and differentiation, which are all related to tumor dormancy. We next focused on the ubiquitination function of ubiquitin-proteasome family of FBX8, by performing Co-IP to screen for downstream interacting proteins. We confirmed that HIF-1α, CDK4, and C-Myc are interactive partners of FBX8 protein. Based on the changes in the expression of HIF-1α, CDK4, and C-Myc in metastatic tumors, we assumed that this regulation is related to ubiquitination degradation. At present, there are many studies on the ubiquitination and degradation function of the F-box family. According to some reports, multiple members of the F-box family can regulate tumor growth and metastasis by participating in ubiquitination degradation of various functional proteins^27^. FBXW7^28^, a recently-identified tumor suppressor, is considered to participate in the ubiquitination and degradation of various proteins, including Mcl-1^29^, Notch^30^, c-Jun^31^, and c-Myc^32^ as well as cyclin E^33^, which are all associated with cell cycle progression. In addition, Fbxs protein member FBXL19 can ubiquitinate and degrade Rac3 to regulate TGF-β1 induced E-cadherin downregulation^34^. The Fbxs protein FBXO7 interacts with CIAP1 (apoptosis inhibitory protein) to promote ubiquitination of CIAP1^35^. In order to accurately assess the ubiquitination degradation function of FBX8, we used the ubiquitin-proteasome inhibitor MG132 to selectively inhibit the proteasome through the cell membrane^36^. Immunofluorescence co-localization assay confirmed that FBX8 could co-localize with HIF-1α, CDK4 and C-myc in CRC cells, and the co-localization was enhanced in the presence of MG132. It has been reported that FBW7 can ubiquitinate and degrade HIF-1α to regulate cell growth, migration, and angiogenesis^37^. c-Myc can promote tumor cell invasion by inhibiting the function of FBX8 through its MBII domain^9^. To further validate the bindings among FBX8 and their substrates, we constructed the mutant forms of FBX8, HIF-1α and C-Myc protein, but CDK4’s full length because of much smaller size. Through the GST-pull-down assay, we found that the GST-FBX-Sec7 domain of FBX8 could directly bind to the HA-HIF-1α-2, 11A-C-Myc-2 truncated sub-region and the full length of HA-CDK4. Subsequent in vitro ubiquitination and half-life assay confirmed that MG132 inhibited the ubiquitination of FBX8 on HIF-1α, CDK4, and Ç-Myc. Thus, we conclude that FBX8 is capable of degrading HIF-1α, CDK4, and C-Myc by direct binding and ubiquitination.

It is well known that, the three ubiquitinated substrates of FBX8, HIF-1α, CDK4 and C-Myc, are closely related to tumor progression. HIF-1α is a nuclear transcription factor that regulates the ability of tumor cells’ adaptation to hypoxia. It also regulates tumor angiogenesis and other functions related to tumor dormancy^38^. CDK4 is the most important protein kinase in the G1 phase of cells and it can synergize with CDK6 and bind to cyclin-D1^39^. CDK4 and other relevant cyclins, including cyclin-D1 and Rb, have extremely close relationship with the progression of many tumors^40^, and the disruption of cell cycle regulation can lead to uncontrolled growth of cells^41^. C-myc regulates dormancy in a variety of tumors by regulating cell cycle, apoptosis, and stemness^42^. Therefore, we performed over-expression and recovery assay on HIF-1α, CDK4, and C-Myc in CRC cells. Microvessel density and lumen formation assay confirmed that the pro-angiogenic ability of tumor cells was restored to some extent after reverse over-expression of HIF-1α. Similarly, CDK4 over-expression promoted the evolution of tumor cell cycle. C-Myc over-expression, as we expected, can promote tumor cell proliferation and inhibit tumor cell apoptosis significantly. FBX8 over-expression enhanced stem potential genetic marker expression of CD133, CD44, and SOX-2. The current prevailing belief is that tumor stem cells and the concept of tumor dormancy can coexist parallelly^43^. Like cancer stem cells, tumor dormant cells also have the ability to proliferate and evade anti-tumor immune responses^44,45^. Many signaling pathways can regulate the growth of cancer stem cells. For example, p38-MAPK, Myc, and TGF-β^46^ can participate in the activation of dormant cells. BMP7 (secreted from bone stromal cells) in the microenvironment can simultaneously regulate dormant tumor cells and cancer stem cells^46^. FBW7 deletion upregulates the number of mouse neural stem cells via Notch and further influence its differentiation^47^. Therefore, dormant tumor cells have many characteristics in common with cancer stem cells. FBX8 not only maintains the stemness of tumor cells but also regulates the biological functions of dormant tumor cells by targeting HIF-1α, CDK4, and C-Myc.

In summary, FBX8 can ubiquitinate and degrade HIF-1α, CDK4 and C-Myc, and downregulate their ability to promote angiogenesis, cell cycle progression and cell proliferation, respectively, thereby regulating the dormancy of CRC liver metastasis cells. The present study reveals a novel mechanism by which FBX8 regulates dormancy in CRC liver metastasis cells, providing a potential target for the treatment of dormant CRC liver metastasis cells as well as offering new theoretical and therapeutic prevention, and treatment strategies to tumor metastasis.

## Supplementary information

Supplementary Figure S1

Supplementary Figure S2

Supplementary Figure S3

Supplementary Figure S4

Supplementary Figure S5

Supplementary Figure S6

Supplementary Figure S7

Supplementary Figure S8

Supplementary information

Supplemental Files-Materials and Methods
