# Supplementary figures and images for: FBX8 promotes metastatic dormancy of colorectal cancer in liver
Source: Cell Death Dis. 2020 Aug 14;11(8):622. doi: 10.1038/s41419-020-02870-7 (PMC7427987; doi:10.1038/s41419-020-02870-7)

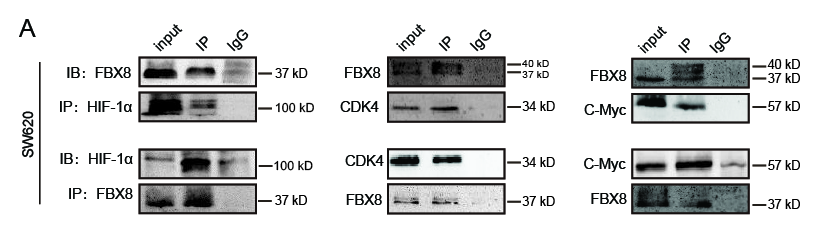

Supplement: Supplementary file 1 — Supplementary Figure S1 [file 41419_2020_2870_MOESM1_ESM.tif]

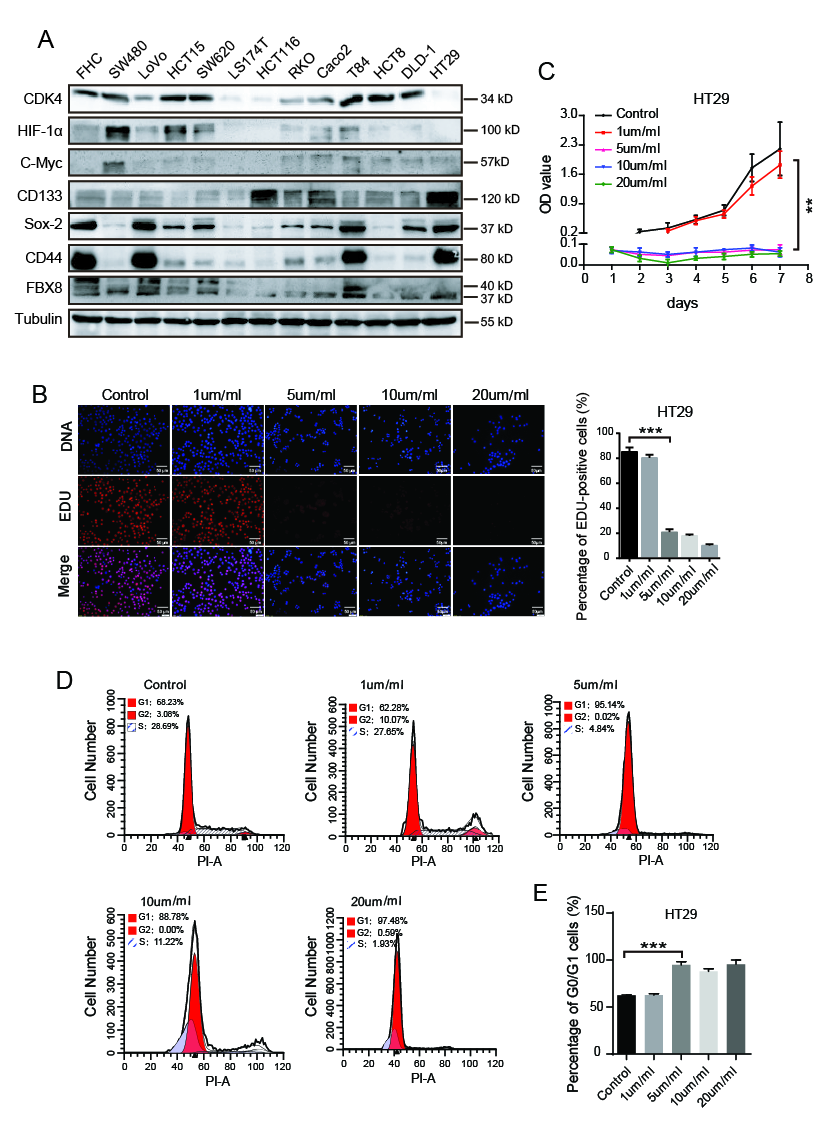

Supplement: Supplementary file 2 — Supplementary Figure S2 [file 41419_2020_2870_MOESM2_ESM.tif]

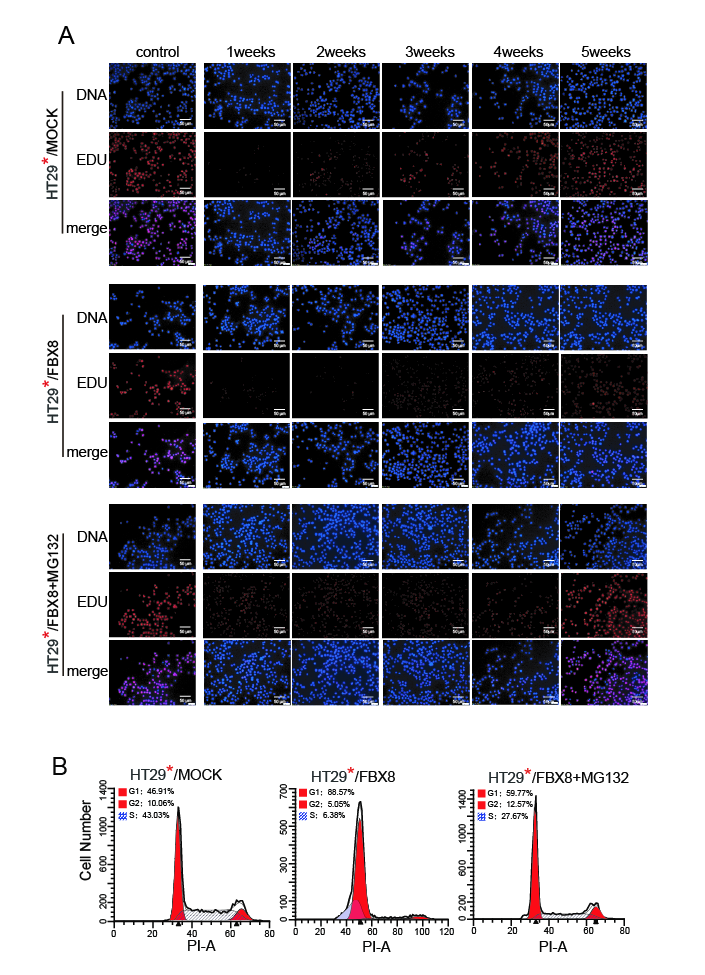

Supplement: Supplementary file 3 — Supplementary Figure S3 [file 41419_2020_2870_MOESM3_ESM.tif]

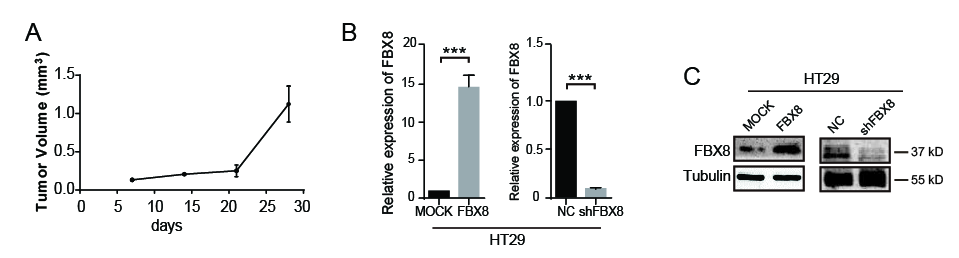

Supplement: Supplementary file 4 — Supplementary Figure S4 [file 41419_2020_2870_MOESM4_ESM.tif]

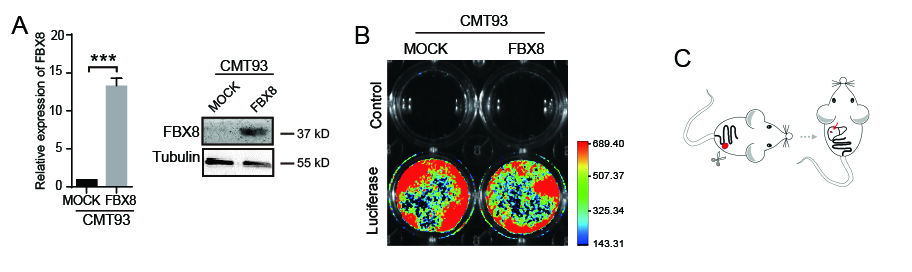

Supplement: Supplementary file 5 — Supplementary Figure S5 [file 41419_2020_2870_MOESM5_ESM.tif]

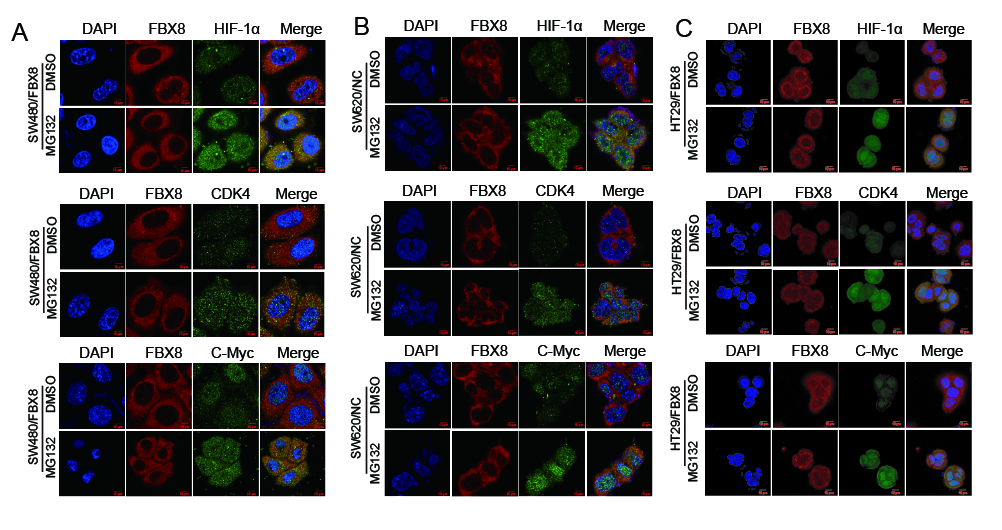

Supplement: Supplementary file 6 — Supplementary Figure S6 [file 41419_2020_2870_MOESM6_ESM.tif]

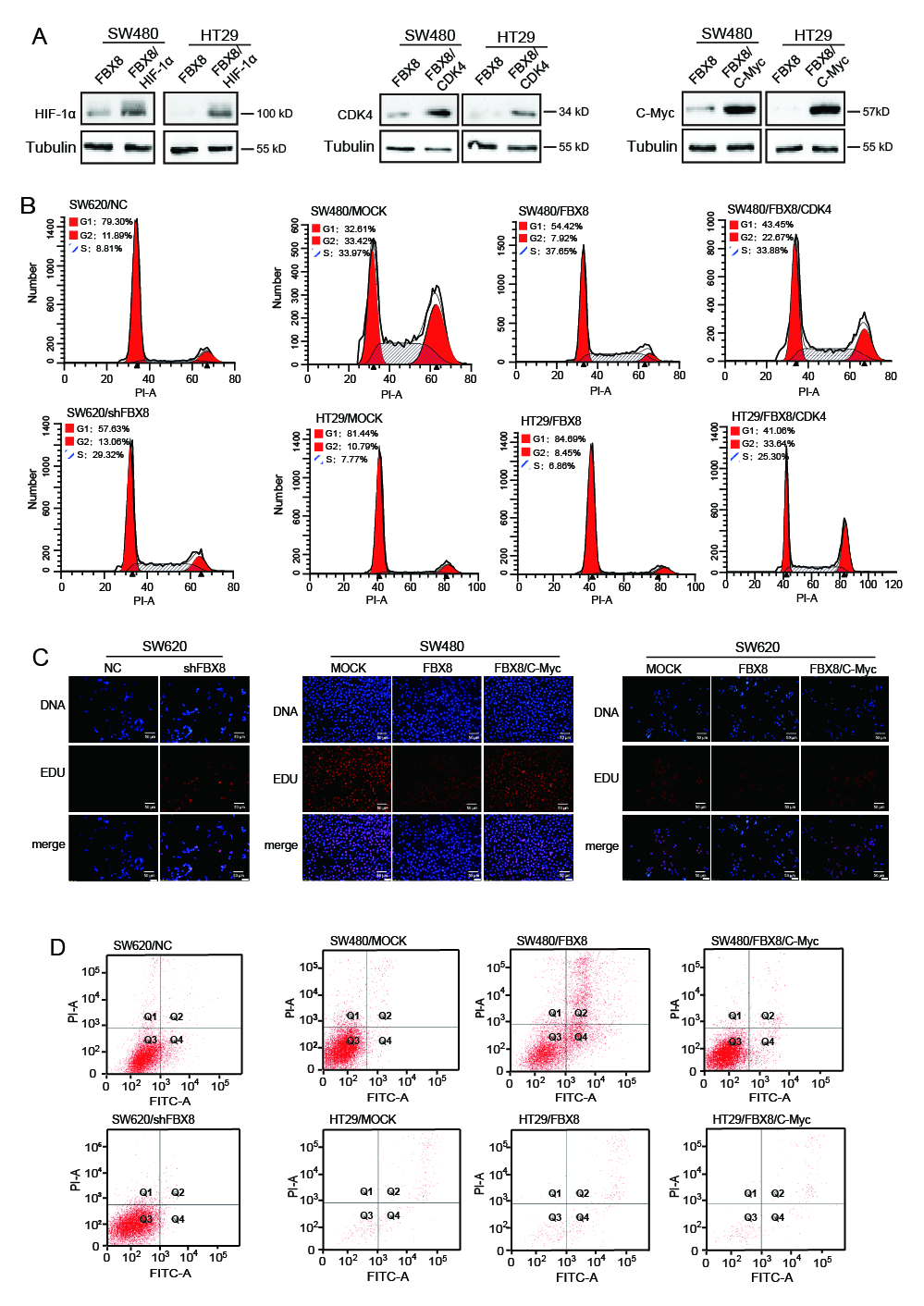

Supplement: Supplementary file 7 — Supplementary Figure S7 [file 41419_2020_2870_MOESM7_ESM.tif]

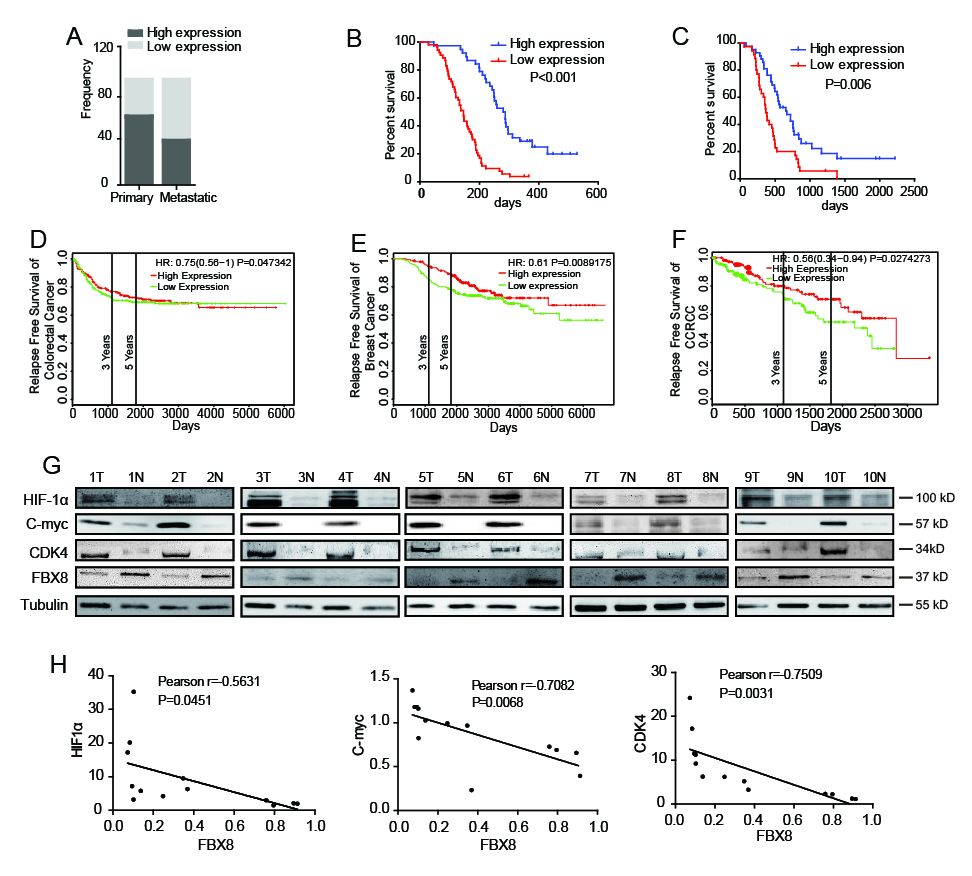

Supplement: Supplementary file 8 — Supplementary Figure S8 [file 41419_2020_2870_MOESM8_ESM.tif]
