# Supplementary material for: FBX8 promotes metastatic dormancy of colorectal cancer in liver
Source: Cell Death Dis. 2020 Aug 14;11(8):622. doi: 10.1038/s41419-020-02870-7 (PMC7427987; doi:10.1038/s41419-020-02870-7)
Supplement: Supplementary file 9 — Supplementary information [file 41419_2020_2870_MOESM9_ESM.docx]

**Supplementary Files-figure legend**

**Figure S1** **The screening of FBX8 interacting proteins by Co-IP assay in SW620 cells.**

**Figure S2 CRC dormancy model induced by combination chemotherapy in vitro**

1. The endogenous expressions of FBX8, CD44, SOX-2, SOX-9, CD133, C-myc, HIF1α and CDK4 in CRC cell lines. (B) Edu analysis was performed for the proliferation of HT29 cells with different concentrations of oxaliplatin and 5-FU combined chemotherapy in vitro. Scale bars represent 50μm. Data represent mean±SD. (C) CCK8 assay was performed for the proliferation of HT29 cells with different concentrations of oxaliplatin and 5-FU combined chemotherapy in vitro. Data represent mean±SD. (D-E) Effects of different concentrations of oxaliplatin and 5-FU combined chemotherapy on HT29 cells proliferation by cell cycle assay. Data represent mean±SD.

**Figure S3 FBX8 inhibits prolifilation of HT29**

1. Edu analysis was performed for the proliferation of HT29 cells with 5μmol/L concentrations of oxaliplatin and 5-FU combined chemotherapy in vitro. Scale bars represent 50μm. (B) Effects of FBX8 on HT29 cells proliferation at 5 weeks by cell cycle assay.

**Figure S4 Growth curve of liver metastasis and construction of stable cell strain**

1. The growth curve of [Liver metastases](C:/Users/11/AppData/Local/youdao/dict/Application/7.5.2.0/resultui/dict/javascript:;) in nude mice. (B)The expression of FBX8 in HT29 cell lines detected by RT-PCR. Data represent mean±SD. (C)The expression of FBX8 in HT29 cell lines detected by Western blot.

**Figure S5 Construction of hepatic metastases dormancy model of C57 mice**

(A) The expression of FBX8 in CMT93 cell lines detected by RT-PCR and Western blot. Data represent mean±SD. (B) The expression of luciferase in CMT93 cell lines. (C) The tumor was surgically removed from the C57 mice cecum on the 7th day after orthotopic injection.

**Figure S6 MG132 inhibits the degradation of HIF-1α, CDK4 and C-Myc by FBX8**

(A-C) Immunofluorescence images of co-localization between FBX8 and HIF-1α, CDK4 and C-Myc in SW480/FBX8, SW620/NC, HT29/FBX8, SW480/FBX8, SW620/NC and HT29/FBX8 cells with MG132 treatment. Scale bars represent 10μm.

**Figure S7 FBX8 promotes CRC dormancy depend on the degradation of HIF-1α, CDK4 and C-Myc**

1. Western blot analyses were performed for HIF-1α, CDK4 and C-Myc in SW480 and HT29 cell lines. (B) The cell cycle of SW620, SW480 and HT29 cell lines. (C) The proliferation of SW620, SW480 and HT29 in vitro detected by Edu. Scale bars represent 50μm. (D) Effects of FBX8 on apoptosis in SW620, SW480 and HT29 cell lines.

**Figure S8 High expression of FBX8 in metastatic carcinoma suggests a good prognosis**

(A) The expression of FBX8 in colorectal cancer tissues and their corresponding hepatic metastases. (B) Kaplan-Meier survival analysis of FBX8 in CRC with lymph node metastasis patients. (C) Kaplan-Meier survival analysis of FBX8 in CRC with liver metastatic patients. (D-F) The Relapse free survival of FBX8 in colorectal cancer, [breast](D:/Dict/7.2.0.0703/resultui/dict/?keyword=breast) [cancer](D:/Dict/7.2.0.0703/resultui/dict/?keyword=cancer) and renal clear cell carcinoma. (G) Western blot analyses of FBX8, HIF1α, CDK4 and C-Myc in 10 paired CRC tissues and corresponding normal tissues. (H) The correlation analysis of FBX8 expression with HIF-1α, CDK4 and c-myc expression in 12 CRC cell lines.

**Supplementary Files-Tables**

**Table S1 List of FBX8, CDK4, HIF1-α, C-Myc and GAPDH primer sequence.**

| FBX8 | FWD | 5’ATGGGTCAAGGGTTGTGGAG 3’ |
| --- | --- | --- |
|  | REV | 5’TTATGCAGCCACATGGCCAA 3’ |
| HIF-1α | FWD | 5’GATCACCCTCTTCGTCGCTT 3’ |
|  | REV | 5’AAAGGCAAGTCCAGAGGTGG 3’ |
| CDK4 | FWD | 5’ATGGCTACCTCTCGATATGAGC 3’ |
|  | REV | 5’CATTGGGGACTCTCACACTCT 3’ |
| C-Myc | FWD | 5’ATGGCCCATTACAAAGCCG 3’ |
|  | REV | 5’TTTCTGGAGTAGCAGCTCCTAA 3’ |
| GAPDH | FWD | 5’ACAGCCCGCAGGATCAGGAAA 3’ |
|  | REV | 5’AACACGCTTCACGGGCACTC 3’ |
