# Supplementary material for: FBX8 promotes metastatic dormancy of colorectal cancer in liver
Source: Cell Death Dis. 2020 Aug 14;11(8):622. doi: 10.1038/s41419-020-02870-7 (PMC7427987; doi:10.1038/s41419-020-02870-7)
Supplement: Supplementary file 10 — Supplemental Files-Materials and Methods [file 41419_2020_2870_MOESM10_ESM.doc]

**Supplemental Files-Materials and Methods**

**Animals, human tissue samples and cell lines**

Four-to-six-week-old male athymic BALB/c-nu/nu mice, BALB/c mice and C57 mice were purchased from the Central Laboratory of Animal Science at Southern Medical University (Guangzhou, China). Each experimental group included six randomly assigned mice. The mice were maintained at our laboratory in a specific Pathogen Free environment. All protocols for animal studies were reviewed and approved by the Institutional Animal Care and Use Committee at our University.

Fresh CRC tissues were collected immediately after resection from 20 patients who underwent CRC resection without prior radiotherapy and chemotherapy at the Department of General Surgery in Nanfang Hospital in 2017 year. Paraffin-embedded CRC primary tumors and liver metastatic tumors samples from 91 patients at Department of Pathology in Nanfang Hospital. For the use of these clinical materials for research purposes, prior patient’s consent and approval from the Institute Research Ethics Committee were obtained.

Human CRC cell lines LoVo, SW480, HCT15, SW620, LS174T, HCT116, RKO, Caco2, T84, HCT8, DLD-1, HT29 and human immortalized colorectal cell line FHC were obtained from Shanghai Cell Bank of Type Culture Collection. The cell lines were freshly bought from ATCC in 2016 year. All cell lines were grown in 1640 medium (GIBCO, Gaithersburg, USA) supplemented with 10% fetal bovine serum (GIBCO, Gaithersburg, USA) at 37℃ under 5% CO2. 5μm/L proteasome inhibitor MG132 (CST) was added in the cultured cells for 24 hours.

MG132 was diluted with DMSO and administered via i.p. at a concentration of 2 mg/kg to each mouse for 7 days running after C57 mice cecal in situ tumor formation.

**Immunohistochemical**

The sections were deparaffinized and rehydrated, and endogenous peroxidase was inhibited with 0.3% H_2_O_2_ methanol. For antigen retrieval, slides were boiled in 0.01M, pH 6.0 sodium citrate buffer for 5 min in a microwave oven. After blocked with the 5% normal goat serum, primary anti-FBX8(1:200), anti-CK(1:200), anti-E-cadherin(1:200), anti-Vimentin (1:200), anti-CDK4(1:200), anti-Sox-9 (1:200), anti-Caspase-3(1:200), anti-Sox-2(1:200), anti-OCT4(1:200), anti-CD44(1:200), anti-CD133(1:200), anti-Ki-67(1:200), anti-cyclinD1(1:200), anti-C-myc(1:200), anti-HIF-1α(1:200) and anti-VEGF(1:200) in blocking buffer were applied and the slides were incubated at 4℃ overnight. After incubation with secondary antibody, the visualization signal was developed with DAB. The stained tissue sections were reviewed and scored separately by two pathologists blinded to the clinical parameters.

**Lentivirus infection**

On the first day, a suitable amount of dormant colorectal cancer HT29 and CT26 cells were placed in a 24-well plate, and 100 ul of complete medium was added, and incubeted in a 37℃, 5% CO _2_ incubator overnight. On the next day, 50 μl of Enhance solution and 0.1 μl of polybrene solution were added to each well. The cells were added to Mock, overexpressed FBX8, NC and shFBX8 lentiviral supernatant, and placed in a 37 ℃, 5% CO_2_ incubator for 8-12 hours. On the third day, the original medium was discarded, and 1640 complete medium was added, and incubation was continued for 72 hours. The fluorescence of the cells was observed under the microscope, the culture was expanded, RNA and whole proteins were extracted, and the expression of FBX8 was detected by Western Blot. The luciferase vector was transferred into CMT93 cells and transferred to Mock and FBX8 lentiviral expression vectors.

**Western blot**

Immunoblotting was performed as follows: Proteins were extracted with a lysis buffer and then quantified by a bicinchoninic acid protein assay. Equivalent amounts of cell lysates were separated using SDS-PAGE and transferred to a polyvinylidene difluoride membrane (Roche Applied Sciences). Membranes were immunoblotted overnight at 4℃ with anti-FBX8 antibodies (Abcom, ab228838, 1:200), anti-CDK4 antibodies（Proteintech, 11026-1-AP, 1:200）、anti-Sox-2 antibodies（Proteintech, 11064-1-AP, 1:200）、anti-Sox-9 antibodies（Proteintech, 67439-1-Ig, 1:200）、anti-OCT4 antibodies（Proteintech, 60242-1-Ig, 1:200）、anti-CD44 antibodies（Proteintech, 15675-1-AP, 1:200）、anti-CD133 antibodies（Proteintech, 66666-1-Ig, 1:200）、anti-Ki-67（Abclone, A11005, 1:200）、anti-cyclinD1 antibodies（Proteintech, 60186-1-Ig, 1:200）、anti-C-myc antibodies（Proteintech, 10828-1-AP, 1:200）、anti-HIF-1α antibodies（Abcam, H1alpha67, 1:200）, anti-GST, anti-HA, anti-Tubulin antibodies (Proteintech, HRP-66001, 51064-2-AP, 11224-1-AP, 1:1000), followed by the appropriate second antibodies. The bands were visualized using Pierce ECL Western Blotting Substrate (Thermo Scientific). Image density of the immunoblotting was determined by Gel densitometry (Bio-Rad).

**Real-time RT-PCR**

Total RNA from the cells and tissue samples was extracted using Trizol reagent (Invitrogen, USA) according to established protocols. The expression level of FBX8, CDK4, HIF1-α and C-Myc was analyzed by quantitative Real-Time PCR (qRT-PCR) using the All-in-One miRNA qRT-PCR Detection Kit (GeneCopeia). The PrimeScript RT Reagent Kit (Perfect Real Time, Takara) was used to generate cDNA for the detection of FBX8, CDK4, HIF1-α and C-Myc. The relative mRNA levels were calculated using the comparative Ct method (ΔΔCt). Primer sequences for qRT-PCR are listed in Supplementary Table S1.

**Luciferase vector transfer efficiency verification**

The luciferase-transferred CMT93 cells were plated in 24-well plates and grew in good condition. A 1:100 luciferase substrate was added and incubated in a 37℃ incubator for 10 min, then photographed using a small animal in vivo imaging system, calculate its fluorescence value.

**Bioinformatics Analysis**

The FBX8 gene was analyzed by the PROGgeneV2, and gene expression was divided into two groups based on the median. Gene enrichment analysis of FBX8 was performed using GSEA data. The data of GSE13294 were collected from 155 patients with primary colorectal cancer. According to the requirements of GSEA software, the data format file was generated, and the data file was uploaded and the corresponding parameters were set to run GSEA.

**Cell proliferation assay(CCK8)**

Medium in 96-well plate was replaced with 100ul mixture (complete medium: CCK8 reagent = 9: 1), incubated at 37 ℃ in 5% CO_2_ for 2-4h. The OD value at 450nm of each well was detected by enzyme labeling instrument, and the OD value was adjusted to zero based on the blank control group. Each experiment was repeated three times. The cell proliferation curve was plotted with the mean value of 5 multiple holes in each group.

**EdU cell proliferation assay**

Prepare EdU working solution (solution A) with RPMI-1640 medium (dilution ratio: 1000:1), The final concentration was 50μM and the medium should be prepared when using. 50μl of EdU-containing RPMI-1640 medium was added to 96-well plate cells, and incubated for 2 hours.

The medium containing EdU was discarded, the cells were washed twice with PBS sterilizated by high temperature and high pressure and PBS was discarded. Each well was added in 500μl cell fixative with 4% paraformaldehyde in PBS solution, fixed at room temperature for 30 min, and the cell fixative were discarded. Then add 50μl l2mg/ml glycine into per well, incubate for 5 min on a shaker at room temperature, discard the glycine, wash twice with 500μl PBS, shake for 5 min each time; discard the PBS solution;

Add 0.5% Triton-X 100μl penetrant prepared in PBS solution to each well, incubate for 10 min on a shaker at room temperature, and wash twice with PBS for 5 min each time;

The Apollo staining reaction solution was prepared in advance, and 50 μl of 1×Apollo staining reaction solution was added to each well, and incubated at room temperature for 30 min on a shaker without light, then discard the staining reaction solution;

Each well was rinsed twice with 500 μl methanol for 5 min and washed twice with PBS for 10 min. Reagent F was diluted with deionized water at a ratio of 100: 1, and a proper amount of 1 × Hoechst33342 reaction solution (currently prepared) was prepared according to the instruction, and stored temporarily in shade, 50 μl of 1 ×Hoechst33342 reaction solution was added into each well, incubated at room temperature for 30 min in shade shaker, and the staining reaction solution was discarded; 500 μl of PBS was adde and washed on a shake table 3 times for 5 min each time.

5 fields of view were randomly selected for each well with the fluorescence microscope. Cells with positive expression and nuclear staining were counted and positive rates were calculated. Each experiment was repeated three times.

**Cell cycle**

10×10^6^ cells of HT29 cells in good condition were collected, washed twice with cold PBS solution, digested with trypsin, centrifuged at 1000 rpm for 5 min, and the cells were resuspended in complete medium and counted. The number of collected cells was about 1×10^5^-1×10^6^ cells. The cells were washed twice with PBS solution, centrifuged at 2000 rpm for 5 min each time, the PBS solution was discarded, and the filter paper was drained. After adding 70% ethanol and fixing at 4℃for 2 h, then centrifuged, and washed twice with the PBS solution, and centrifuged at 2000 rpm for 5 min. Add 100ul of RNase A and place it in a 37℃ water bath for 30min, then add PI dye solution to 400ul and mix well. Protect from light and incubate at 4℃ for 30min. After dyeing, it should be tested within 1h and record the red fluorescence at 488nm. Each experiment was repeated three times.

**Apoptosis**

10×10^6^ cells of HT29 cells were collected, washed twice with PBS solution, digested with trypsin without EDTA, resuspended after centrifugation, then counted and about 1×10^5^-1×10^6^ cells were collected. The cells were washed twice with PBS solution, centrifuged at 2000 rpm for 5 min each time, the PBS solution was discarded, and the filter paper was drained. Add Binding-buffer 500μl resuspended cells to each tube, then add 5μl of Annexin V reagent, mix thoroughly by pipetting, and finally add 5μl of Propidium Iodide reagent, mix thoroughly by pipetting, incubate at room temperature for 5-15min. After dyeing, the apoptosis was measured by flow cytometry within 1 h. Each experiment was repeated three times.

**Drug treatment**

SW480/Mock, SW480/FBX8, SW620/NC and SW620/shFBX8 cells were plated into a 6-well plate for culture. When the cells were fused to about 85%, the protein synthesis inhibitor cycloheximide (CHX) was added. The SW480/FBX8 and SW620/NC groups were simultaneously added to the MG132. Cellular proteins were collected at 0h, 12h, 24h, 36h, 48h, and 60h after drug treatment.

**Statistical analysis**

The data of different groups of CCK8 were designed by factorial analysis; the two samples were compared by independent T test; the mean of cell cycle, apoptosis and EDU were compared by one-way ANOVA. Before the analysis of variance, Levene test was used for variance. Homogeneity test, LSD method was used for multiple comparisons of variances; Dunnett's 3 method was used for multiple comparisons when variance was not used; Pearson correlation analysis was used for correlation of endogenous detection. C57 mouse colorectal cancer liver metastasis sleep model liver metastasis cancer size was analyzed by one-way repeated measures data analysis of variance.
